# AMPK deficiency in chondrocytes accelerated the progression of instability-induced and ageing-associated osteoarthritis in adult mice

**DOI:** 10.1038/srep43245

**Published:** 2017-02-22

**Authors:** Sheng Zhou, Wanli Lu, Liang Chen, Qiting Ge, Dongyang Chen, Zhihong Xu, Dongquan Shi, Jin Dai, Jianxin Li, Huangxian Ju, Yi Cao, Jinzhong Qin, Shuai Chen, Huajian Teng, Qing Jiang

**Affiliations:** 1 Department of Sports Medicine and Adult Reconstructive Surgery, Drum Tower Hospital, School of Medicine, Nanjing University, 321 Zhongshan Road, Nanjing 210008, Jiangsu, P.R. China; 2 Laboratory for Bone and Joint Disease, Model Animal Research Center (MARC), Nanjing University, Nanjing 210093, Jiangsu, China; 3 State Key Laboratory of Analytical Chemistry for Life Science, Nanjing University, Hankou Road, Nanjing 210093, Jiangsu, China; 4 Collaborative Innovation Center of Advanced Microstructures, National Laboratory of Solid State Microstructure and Department of Physics, Nanjing University, Hankou Road, Nanjing 210093, Jiangsu, China

## Abstract

Osteoarthritis (OA) is a progressive degenerative disease of the joints that is associated with both joint injury and ageing. Here, we investigated the role of the energy sensor AMP-activated protein kinase (AMPK) in maintaining a healthy state of articular cartilage and in OA development. Using cartilage-specific, tamoxifen-inducible *AMPKα1* conditional knockout (*AMPKα1* cKO), *AMPKα2* conditional knockout (*AMPKα2* cKO) and *AMPKα1α2* conditional double knockout (*AMPKα* cDKO) mice, we found that compared with wild-type (WT) littermates, mutant mice displayed accelerated severity of surgically induced OA, especially *AMPKα* cDKO mice. Furthermore, male but not female *AMPKα* cDKO mice exhibited severely spontaneous ageing-associated OA lesions at 12 months of age. The chondrocytes isolated from *AMPKα* cDKO mice resulted in an enhanced interleukin-1β (IL-1β)-stimulated catabolic response. In addition, upregulated expression of matrix metalloproteinase-3 (MMP-3), MMP-13 and phospho-nuclear factor-κB (phospho-NF-κB) p65 and increased levels of apoptotic markers were detected in the cartilage of *AMPKα* cDKO mice compared with their WT littermates *in vivo*. Thus, our findings suggest that AMPK activity in chondrocytes is important in maintaining joint homeostasis and OA development.

Osteoarthritis (OA) is the most common joint disease and the major cause of disability in aged individuals. Multiple factors contribute to OA development, such as hereditary, ageing and mechanical stress^1^,^2^. Although great efforts have been made, there are still no effective disease-modifying OA therapies to date partly because the exact mechanism of OA pathogenesis is still not fully understood.

AMP-activated protein kinase (AMPK) is an evolutionarily conserved serine/threonine kinase and exists as heterotrimeric complexes containing one catalytic subunit (encoded by α1 or α2) and two regulatory β and γ subunits^3^,^4^. Phosphorylation at Thr-172 within the catalytic α subunit is a prerequisite for AMPK activation^3^,^4^. Once activated, AMPK phosphorylates various downstream substrates, allowing the inhibition of energy (*e.g*., ATP) consuming cellular processes and the activation of energy-producing processes^4^. In this manner, AMPK is a master regulator of energy homeostasis, and AMPK dysregulation has been implicated in diverse human diseases and ageing^5^,^6^,^7^.

AMPK activity is constitutively present in normal articular chondrocytes^8^. There is evidence that reduced AMPK activity in chondrocytes is associated with OA and senescence^8^,^9^,^10^. Reduced AMPKα phosphorylation was noted in mouse surgical instability-induced and human OA knee cartilage^8^,^9^. AMPKα phosphorylation in aged mouse knee cartilage was also reduced^9^,^10^. Interleukin-1β (IL-1β) or tumour necrosis factor α (TNFα) downregulates the activity of AMPK in chondrocytes, and upregulating AMPK activity attenuated IL-1β and TNFα-induced catabolic gene expression in chondrocytes *in vitro*^8^,^9^. Consistently, AMPK pharmacological activators exerted a chondroprotective effect *in vivo*^11^,^12^,^13^. AMPK is an emerging regulator of the inflammatory process in OA^14^,^15^,^16^. However, a recent study reported that chondrocyte-specific ablation of *AMPKα1* failed to affect OA pathogenesis in a surgically induced OA mouse model^17^. In the present study, adult cartilage-specific *AMPKα1* conditional knockout, *AMPKα2* conditional knockout and *AMPKα1α2* conditional double knockout mice were generated. The accurate effect of AMPK on the maintenance of adult articular cartilage in OA pathogenesis *in vivo* and its underlying mechanisms were assessed. Our results indicate that *AMPK* deficiency in chondrocytes disrupts articular cartilage homeostasis in adults by enhancing catabolic activity and promoting chondrocyte apoptosis in surgery-induced and ageing-associated OA.

## Results

### AMPKα1α2 recombination in chondrocytes

**Figure 1:**
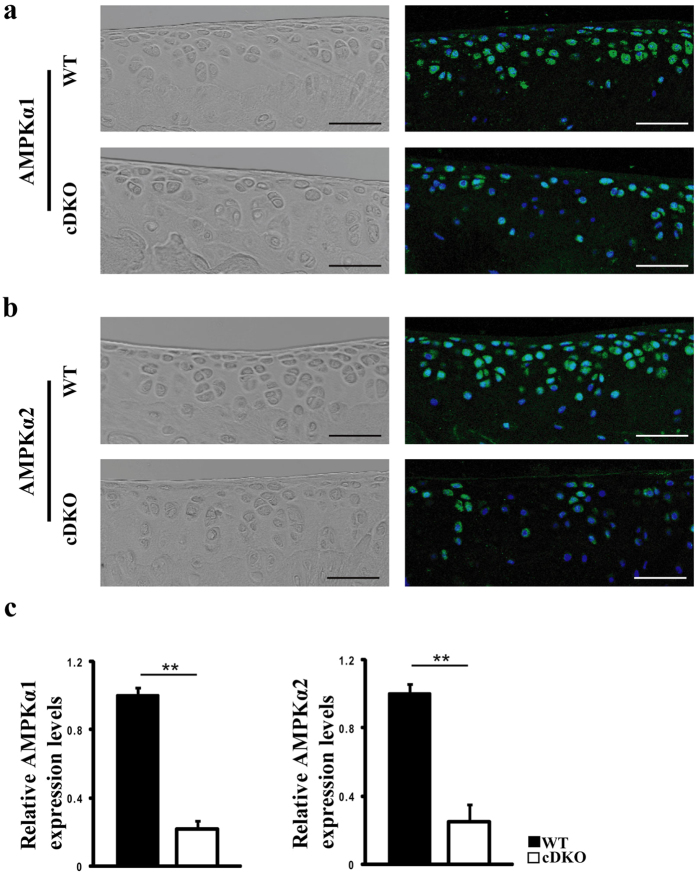
Efficiency of AMPK ablation in chondrocytes. (**a**) Immunofluorescence analysis showed that AMPKα1 and AMPKα2 expressions levels were reduced in articular chondrocytes from 10-week-old *AMPKα* cDKO mice treated with TM at age 8 weeks for 5 days compared with the levels in their WT littermates (n = 6/group). Green indicates positive staining. Blue indicates DAPI staining. Scale bars = 50 μm. (**b**) Real-time reverse transcriptase-PCR analysis reveals significant reductions in *AMPKα1* and *AMPKα2* messenger RNA (mRNA) expression levels in cartilage obtained from 10-week-old *AMPKα* cDKO mice compared with the levels in their WT littermates (n = 6/group). These mice were treated with TM at age 8 weeks for 5 days. **p < 0.01.

We generated tamoxifen (TM)-inducible and cartilage-specific *AMPKα1* conditional knockout, *AMPKα2* conditional knockout and *AMPKα1α2* conditional double knockout mice (treated with TM at 8 weeks of age, 0.1 mg/g body weight/day for 5 days). *Col2a1-CreER*^*T2*^; *AMPKα1*^*flox/flox*^, *Col2a1-CreER*^*T2*^; *AMPKα2*^*flox/flox*^ and *Col2a1-CreER*^*T2*^; *AMPKα1*^*flox/flox*^*α2*^*flox/flox*^ mice treated with TM at 8 weeks of age are hereafter referred as to *AMPKα1* cKO, *AMPKα2* cKO and *AMPKα* cDKO mice, respectively. *AMPKα1, AMPKα2* and *Cre* transgene were genotyped by PCR in these mice (Supplementary Fig. S1a). Immunofluorescence (IF) analysis showed remarkably reduced AMPKα1 and AMPKα2 protein expression in the articular cartilage of tibial plateaus of 10-week-old *AMPKα* cDKO mice (n = 6/group; Fig. 1a,b). Apparent decreases in the messenger RNA (mRNA) expression levels of *AMPKα1* and *AMPKα2* in the articular cartilage of 10-week-old *AMPKα* cDKO mice were confirmed by q-PCR (n = 6/group; Fig. 1c; Unpaired *t*-test; *AMPKα1*, p = 0.005; *AMPKα2*, p = 0.005).

### Disruption of AMPK during the adult stage did not lead to basal cartilage abnormalities

**Figure 2:**
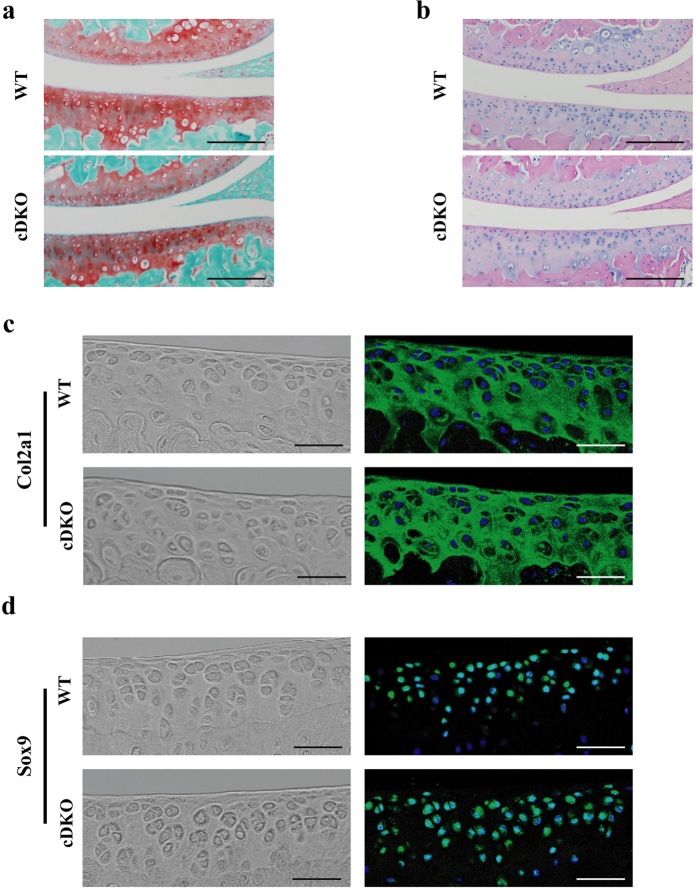
Basal articular cartilage in *AMPKα1α2* conditional double knockout (*AMPKα* cDKO) mice and their Cre-negative wild-type (WT) littermates. (**a**) Safranin-O/Fast green and (**b**) H&E staining of knee joints from 10-week-old *AMPKα* cDKO mice and their WT littermates administrated TM at age 8 weeks for 5 days (n = 6/group). Scale bars = 100 μm. Representative IF images of (**c**) Col2a1 and (**d**) Sox9 in knee joints from 10-week-old *AMPKα* cDKO mice and their WT littermates administrated TM at age 8 weeks for 5 days (n = 6/group). Green indicates positive staining. Blue represents DAPI staining. No intensity or background adjustments were made between sections. Col2a1 = Type II collagen. Scale bars = 50 μm.

We then examined the characteristics of the articular cartilage of 10-week-old mice. No gross abnormalities in knee joints and no significant structural changes or proteoglycans losses in articular cartilage were observed in either *AMPKα* cDKO mice or their wild-type (WT) littermates (Fig. 2a,b). The growth plate width of *AMPKα* cDKO mice was similar to those of their WT littermates. *AMPKα* cDKO mice expressed Col2a1 and Sox9 at levels comparable to their WT littermates in the articular cartilage (Fig. 2c,d). As expected, both 10-week-old *AMPKα1* cKO and *AMPKα2* cKO males exhibited articular cartilage and growth plate characteristics that were similar to their WT littermates.

### Exacerbated OA in AMPK mutant mice following surgical destabilization of the knee

**Figure 3:**
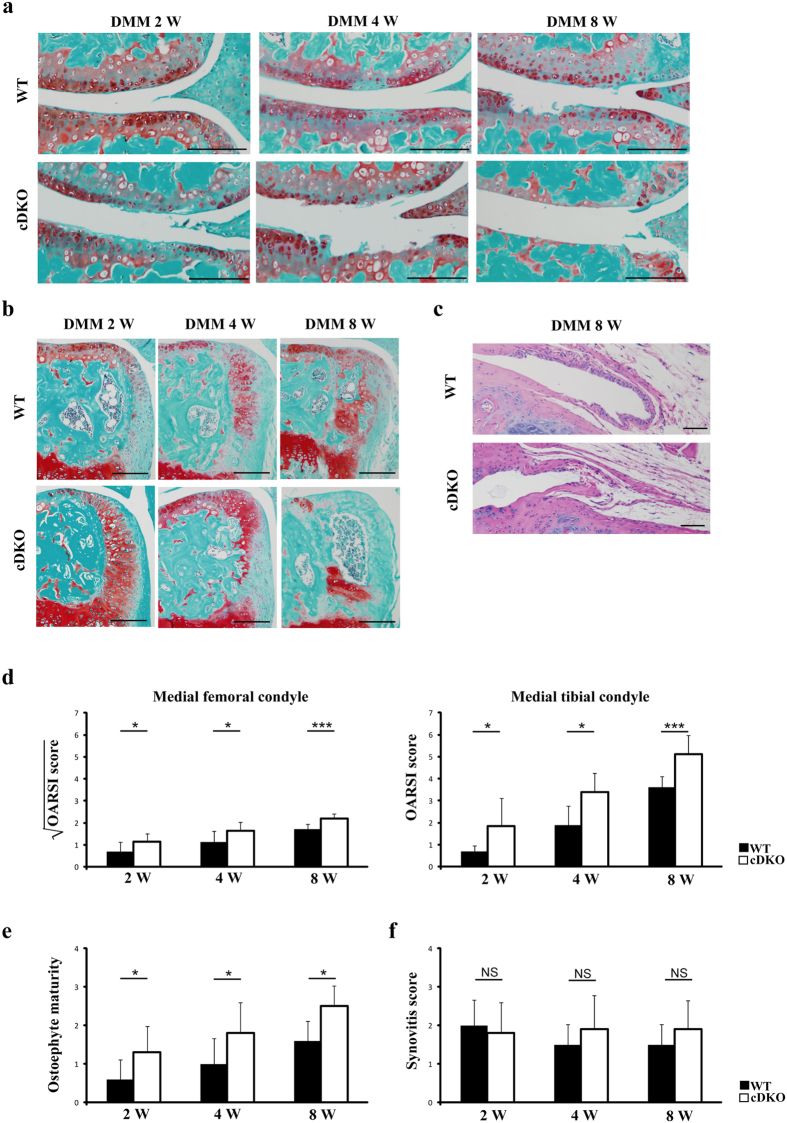
Accelerated OA in *AMPKα1α2* conditional double knockout (*AMPKα* cDKO) mice following destabilization of the medial meniscus (DMM). Representative photographs of articular cartilage destruction (**a**) or osteophyte formation (**b**) in *AMPKα* cDKO mice and their WT littermates 2, 4 and 8 weeks post-DMM (n = 10/group). Sections were stained with Safranin O/Fast Green. Scale bars = 100 μm. (**c**) Representative photographs of synovitis in *AMPKα* cDKO mice (n = 10) and their WT littermates (n = 10) at 8 weeks post-DMM. Sections were stained with H&E. Scale bars = 50 μm. The OARSI scores for the medial femoral condyle and the medial tibial condyle (**d**) and osteophyte maturity (**e**) at 2, 4 and 8 weeks post-DMM, and synovitis scores (**f**) at 8 weeks post-DMM in *AMPKα* cDKO mice and their WT littermates (n = 10/group). The OARSI scores for the medial femoral condyle of *AMPKα* cDKO and their WT littermates were transformed by taking the square root of the values. After transformation, all groups of data approximate a Gaussian distribution. *p < 0.05. **p < 0.01. ***p < 0.001. NS = not significant.

No obvious abnormalities in joint morphology were noted in 10-week-old *AMPKα1* cKO, *AMPKα2* cKO and *AMPKα* cDKO mice compared with WT littermates, indicating that these mice are suitable for OA studies. Therefore, we analysed the development of instability-induced OA changes in *AMPK* mutant and WT mice using the destabilization of the medial meniscus (DMM) model as previously described^18^. DMM is a progressive osteoarthritis model characterized by articular cartilage destruction, osteophyte formation and little or no synovitis. Safranin-O/Fast green staining of cartilage in WT mice demonstrated that cartilage destruction slowly progressed into the middle zone by 4 weeks post-DMM and reached the calcified cartilage layer by 8 weeks (Fig. 3a). *AMPKα* cDKO mice were more responsive to instability-induced OA progression than were their WT littermates. Roughening of the articular cartilage, loss of proteoglycans and chondrocyte cellularity were noted at 2 weeks post-surgery, whereas complete loss of the entire articular cartilage and exposed subchondral bone were noted at 8 weeks post-DMM in both the medial tibial plateau and the medial femoral condyle (Fig. 3a). The cartilage destruction in *AMPKα1* cKO and *AMPKα2* cKO mice was comparable to their WT littermates at 2 and 4 weeks post-DMM, but they exhibited more severe OA-like phenotypes, including loss of uncalcified cartilage, a reduced number of chondrocytes, and alteration of the tidemark integrity (indicated by the arrowheads in Supplementary Fig. S2a,c). To quantify the severity of the cartilage damage, we compared the OARSI scores of articular cartilage histologic structure in either *AMPK* mutant mice or their WT littermates 2, 4 and 8 weeks post-DMM. The scores for morphological structure changes in the medial femur and the medial tibia were significantly greater in knee joints from *AMPKα* cDKO mice than in those of their WT littermates at all time points examined post-DMM (Fig. 3d; 2-way ANOVA; Femur: 2 W, p = 0.027; 4 W, p = 0.023; 8 W, p < 0.001; Tibia: 2 W, p = 0.021; 4 W, p = 0.047; 8 W, p < 0.001). However, OA scores were significantly increased in *AMPKα1* cKO and *AMPKα2* cKO mice compared with those in the WT littermates at 8 weeks, but not at 2 or 4 weeks, post-surgery (Supplementary Fig. S2b,d; 2-way ANOVA; *AMPKα1* cKO mice Femur: 8 W, p = 0.010; Tibia: 8 W, p = 0.003; *AMPKα2* cKO mice Femur: 8 W, p = 0.003; Tibia: 8 W, p = 0.027). Slightly progressive cartilage damage was observed in the contralateral, sham-operated knees of *AMPKα* cDKO mice 8 weeks after DMM surgery. This damage was significantly greater than in the WT littermates at 8 weeks (Supplementary Fig. S3a; 2-way ANOVA; Femur: 8 W, p = 0.045; Tibia: 8 W, p = 0.001). However, sham-operated knees from *AMPKα1* cKO or *AMPKα2* cKO mice exhibited no obvious cartilage damage (Supplementary Fig. S3b,c).

The joints in *AMPKα* cDKO mice exhibited increased cartilaginous outgrowth and osteophyte formation 2, 4 and 8 weeks post-DMM (Fig. 3b), whereas osteophyte formation in *AMPKα1* cKO and *AMPKα2* cKO mice was indistinguishable from that in their WT littermates at these three times post-surgery. These observations were corroborated by the osteophyte maturity scores (Fig. 3e; 2-way ANOVA; 2 W, p = 0.035; 4 W, p = 0.030; 8 W, p = 0.020). No significant differences were observed in synovitis scores when comparing *AMPKα1* cKO, *AMPKα2* cKO, *AMPKα* cDKO mice with their WT littermates 2, 4 and 8 weeks post-DMM (Fig. 3c,f). Sham-operated knees from mice of either genotype did not exhibit obvious osteophyte formation or synovial inflammation.

### Exacerbated OA in aged AMPK mutant mice

**Figure 4:**
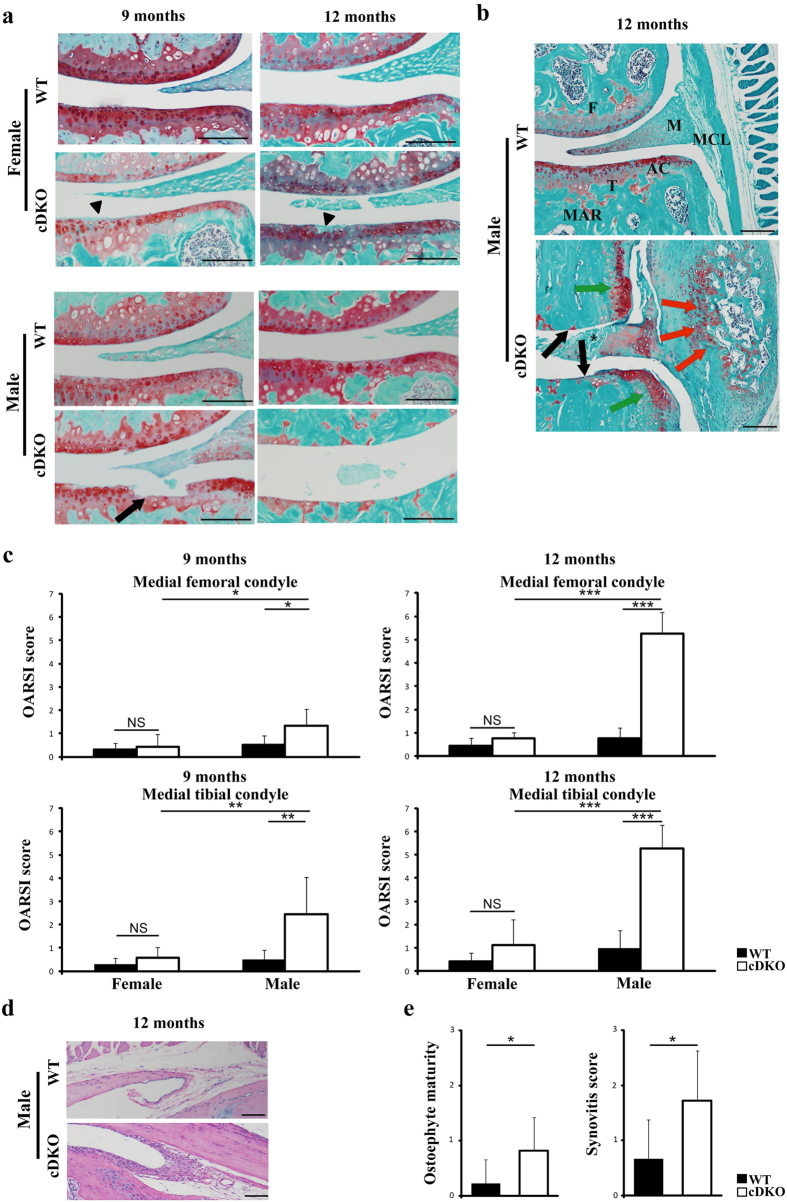
Accelerated OA in *AMPKα1α2* conditional double knockout (*AMPKα* cDKO) mice with ageing. (**a**) Representative photographs of articular cartilage destruction in female and male *AMPKα* cDKO mice and their age- and sex- matched WT littermates at 9 and 12 months of age. Sections were stained with Safranin O/Fast Green. A slight loss of proteoglycans in the superficial zone and fibrillation in articular cartilage (arrowhead) were observed in female *AMPKα* cDKO mice at 9 or 12 months. Loss of uncalcified cartilage and alteration of the tidemark integrity (arrow) were observed in male *AMPKα* cDKO mice at 9 months. Scale bars = 100 μm. (**b**) Representative Safranin-O/Fast green staining of knee joints from male *AMPKα* cDKO mice and their sex matched WT littermates at 12 months of age. Loss of the entire articular cartilage layer (black arrows), formation of osteophyte (green arrows), severe disruption of meniscal tissue (asterisk) and bone marrow-like regions in the enlarged medial collateral ligament (red arrows) were observed in male *AMPKα* cDKO mice at 12 months of age. T, tibia; F, femur; AC, articular cartilage; M, meniscus; MCL, medial collateral ligament. MAR, marrow cavities. Scale bars = 100 μm. (**c**) The OARSI scores for the medial femoral condyle and the medial tibial condyle in female and male *AMPKα* cDKO mice and their sex-matched WT littermates at 9 and 12 months of age (9 months old: WT females n = 5, cDKO females n = 7, WT males n = 7, cDKO males n = 9; 12 months old: WT females n = 7, cDKO females n = 8, WT males n = 8, cDKO males n = 11). (**d**) Representative photographs of synovitis in male *AMPKα* cDKO mice and their sex-matched WT littermates at 12 months of age. Sections were stained with H&E. Scale bars = 50 μm. (**e**) Osteophyte maturity and synovitis scores in male *AMPKα* cDKO mice (n = 11) and their sex-matched WT littermates (n = 8) at 12 months of age. *p < 0.05. **p < 0.01. ***p < 0.001. NS = not significant.

Spontaneous degeneration of articular cartilage in female or male *AMPKα1* cKO, *AMPKα2* cKO or *AMPKα* cDKO mice at 9 or 12 months of age was histologically assessed and compared with their age- and sex-matched WT littermates. In both genders, *AMPKα1* cKO and *AMPKα2* cKO mice exhibited OA scores, synovial inflammation and osteophyte formation similar to their WT littermates at the ages of 9 or 12 months. The female *AMPKα* cDKO mice at 9 or 12 months displayed a slight loss of proteoglycans in the superficial zone and fibrillation in articular cartilage (indicated by the arrowhead in Fig. 4a). In contrast, *AMPKα* cDKO males at the age of 9 months showed obvious incipient articular cartilage degradation, such as loss of uncalcified cartilage and alteration of the tidemark integrity (indicated by the arrow by Fig. 4a), and more severe joint lesions were observed in mice at the age of 12 months (Fig. 4a). OARSI scores were significantly increased in the medial femoral condyles and the medial tibial plateaus of 9 and 12-month-old *AMPKα* cDKO males compared with their age- and sex-matched WT littermates (Fig. 4c; 2-way ANOVA; Femur, 9-month-old, p = 0.047; 12-month-old, p < 0.001; Tibia, 9-month-old, p = 0.003; 12-month-old, p < 0.001). No differences in OARSI scores were noted between males and females of the WT group, whereas *AMPKα* cDKO males had higher OARSI scores than *AMPKα* cDKO females (Fig. 4c; 2-way ANOVA; 9-month-old, Femur, p = 0.021; Tibia, p = 0.004; 12-month-old, Femur, p < 0.001; Tibia, p < 0.001). In addition to the severe destruction of articular cartilage, osteophyte formation, damage to the meniscus and enlarged medial collateral ligament with peripheral marrow cavities were observed in 12-month-old *AMPKα* cDKO males (Fig. 4b). Quantifications of osteophyte maturity and inflammatory scores revealed increased values for 12-month-old mice compared with those of their sex-matched WT littermates (Fig. 4d,e; Unpaired *t*-test; osteophyte maturity, p = 0.010; synovitis score, p = 0.010). Taken together, our findings indicate that mice with *AMPKα* disruption are prone to developing spontaneous ageing-associated OA, especially male mice.

### Chondrocyte-specific deletion of AMPKα enhances the procatabolic response to IL-1β *in vitro*

**Figure 5:**
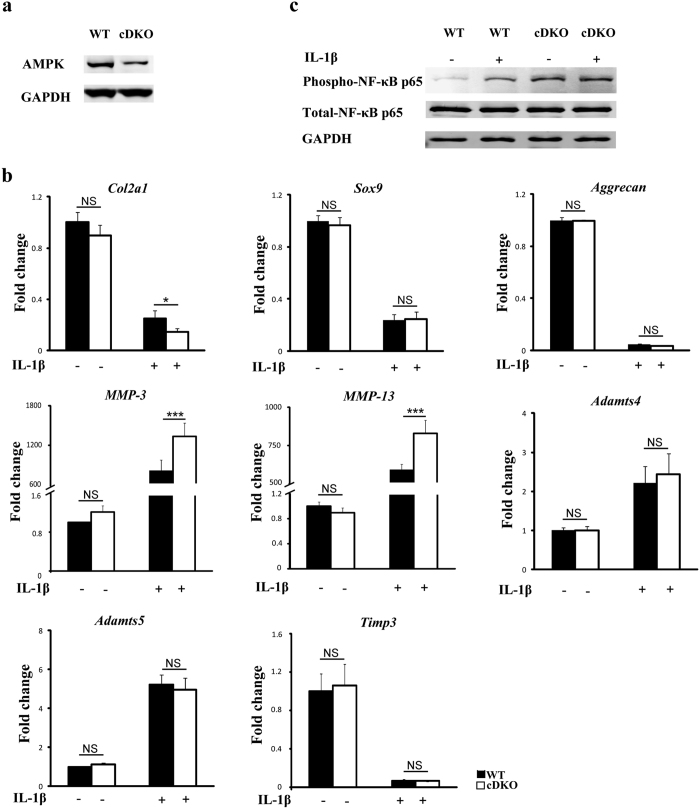
*AMPKα* deficiency enhancing phospho-NF-κB p65 and the procatabolic response to iterleukin-1β (IL-1β) in primary murine chondrocytes. Primary articular chondrocytes were isolated from *Col2a1-CreER*^*T2*^; *AMPKα1*^*flox/flox*^*α2*^*flox/flox*^ mice and their *Cre*-negative WT littermates and treated with 4-hydroxytamoxifen for 48 hours as described in the Methods. (**a**) Western blotting analyses of AMPKα expression. (**b**) The expression levels of *Col2a1, Aggrecan, Sox9, MMP-3, MMP-13, Adamts4, Adamts5* and *Timp3* messenger RNA (mRNA) in primary murine chondrocytes treated with or without IL-1β (10 ng/ml) for 24 h were determined by real-time reverse transcriptase-PCR. Data are representative of three individual experiments. *Col2a1* = Type II collagen; *MMP-3* = matrix metalloproteinase-3; *MMP-13* = matrix metalloproteinase-13; *p < 0.05. ***p < 0.001. NS = not significant. (**c**) Western blotting analyses of total NF-κB p65 and phospho-NF-κB p65 in primary murine chondrocytes with or without IL-1β (10 ng/ml) for 24 h. Increased phospho-NF-κB p65 protein expression in chondrocytes from *AMPKα* cDKO mice compared with their WT littermates was observed. GAPDH served as a loading control.

To further explore the mechanism underlying accelerated OA progression in *AMPKα* cDKO mice, we first performed *in vitro* experiments. Western blotting confirmed the deletion of the *AMPKα* gene in chondrocytes derived from *Col2a1-CreER*^*T2*^; *AMPKα1*^*flox/flox*^*α2*^*flox/flox*^ mice (Fig. 5a). Isolated primary chondrocytes from 5-day-old *AMPKα*-deficient mice and their *Cre*-negative littermates were cultured with or without IL-1β for analysis. Q-PCR analysis revealed that the basal expression levels of selected anabolic genes (*Col2a1, Aggrecan* and *Sox9*) and catabolic genes (*MMP-3, MMP-13, Adamts4* and *Adamts5*) were not significantly different in *Col2a1-CreER*^*T2*^; *AMPKα1*^*flox/flox*^*α2*^*flox/flox*^ mouse chondrocytes than those in WT mouse chondrocytes (Fig. 5b). Next, we treated primary articular chondrocytes with IL-1β. Reduced *Col2a1* levels and increased *MMP-3* and *MMP-13* levels via IL-1β were significantly enhanced in chondrocytes from *AMPKα*-deficient mice, whereas reduced *Sox9, Aggrecan* and *Timp3* levels and increased *Adamts4* and *Adamts5* levels were not significantly different between the two groups (Fig. 5b; 1-way ANOVA; *Col2a1*, p = 0.042; *MMP-3*, p < 0.001; *MMP-13*, p < 0.001). Given the role of the NF-κB signalling pathway in mediating the inflammatory process, such as matrix metalloproteinases (MMPs)^19^,^20^, we further explored the phosphorylation of the nuclear factor-κB (NF-κB) p65 subunit at Ser536. IL-1β induced more phosphorylation of the NF-κB p65 protein in a primary culture of *AMPKα*-deficient chondrocytes than in WT chondrocytes (Fig. 5c). Taken together, these results demonstrate that *AMPKα* deficiency in chondrocytes enhanced the induction of catabolic genes (*MMP-3* and *MMP-13*) by proinflammatory stimuli (IL-1β) with negligible effects on the basal expression levels of anabolic or catabolic genes.

### Increased expression of MMP-3, MMP-13, phospho-NF-κB-p65 and apoptotic marker in *AMPKα* cDKO mice *in vivo*

**Figure 6:**
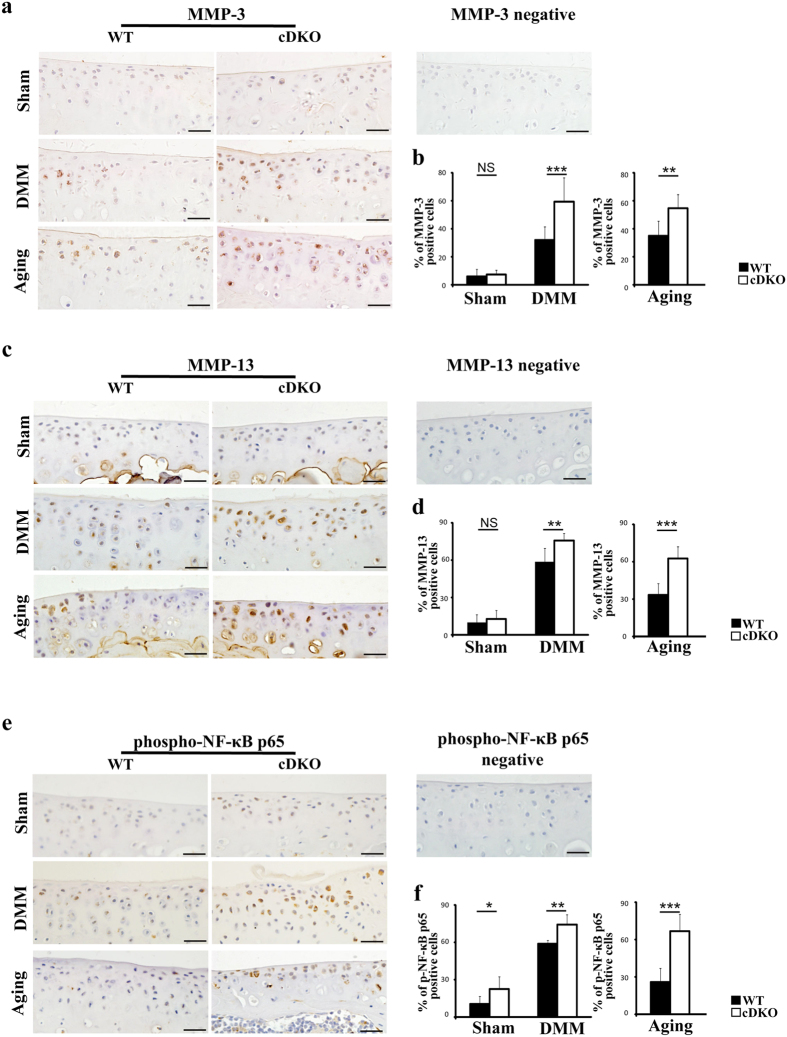
IHC analyses of surgically induced and ageing-associated OA. (**a**,**c** and **e**) Representative IHC images of MMP-3, MMP-13 and phospho-NF-κB p65 in the medial tibial plateau in *AMPKα1α2* conditional double knockout (*AMPKα* cDKO) mice and their WT littermates 2 weeks post-sham operation and DMM surgery or in mice at 9 months of age. Scale bars = 20 μm. The cellularity of the section was confirmed with haematoxylin staining. (**b**,**d**,**f**) Quantifications of the percentages of MMP-3, MMP-13 and phospho-NF-κB p65 are presented as percentages relative to cells stained for haematoxylin. (n = 6/group) MMP-3 = matrix metalloproteinase-3; MMP-13 = matrix metalloproteinase-13. *p < 0.05. **p < 0.01. ***p < 0.001. NS = not significant.

**Figure 7:**
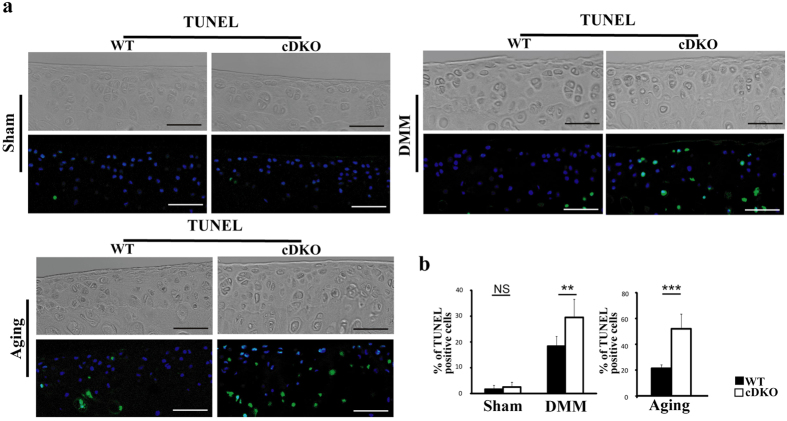
TUNEL analyses of surgically induced and ageing-associated OA. (**a**) Representative TUNEL images in the medial tibial plateau in *AMPKα1α2* conditional double knockout (*AMPKα* cDKO) mice and their WT littermates 2 weeks post-sham operation and DMM surgery or in mice at 9 months of age. Green represents positive staining. Blue indicates DAPI staining. No intensity or background adjustments were made between sections. Scale bars = 50 μm. (**b**) Quantifications of the percentage of TUNEL-positive cells are presented as percentages relative to cells stained for DAPI. (n = 6/group) TUNEL = terminal deoxynucleotidyl transferase dUTP nick end labelling; **p < 0.01. ***p < 0.001. NS = not significant.

The hallmark of OA is an imbalance between chondrocyte anabolism and catabolism and increased chondrocyte apoptosis. To better understand the mechanism underlying the accelerated OA progression in *AMPKα* cDKO mice *in vivo*, we conducted immunohistochemistry (IHC) and terminal deoxynucleotidyl transferase dUTP nick end labelling (TUNEL) analyses. Changes in Adamts4, Adamts5, Timp3, MMP-3, MMP-13, phospho-NF-κB p65 and chondrocyte death were evaluated 2 weeks post-surgery and at the age of 9 months (at these two time points, the cartilage was not severely damaged in *AMPKα* cDKO mice). Expression levels of the cartilage-degrading enzymes MMP-3 and MMP-13 were significantly increased in *AMPKα* cDKO mice compared with those in their WT littermates 2 weeks after DMM surgery and at 9 months, whereas MMP-3 and MMP-13 expression levels were not significantly different between the sham operation group of *AMPKα* cDKO and WT mice (Fig. 6a–d; MMP3, 1-way ANOVA; DMM, p < 0.001; Unpaired *t*-test; Ageing, p = 0.008; MMP-13, 1-way ANOVA; DMM, p = 0.006; Unpaired *t*-test; Ageing, p < 0.001). Significant increases in the percentages of phospho-NF-κB p65-positive cells were observed in knee cartilage 2 weeks both post-sham operation and DMM group from *AMPKα* cDKO mice compared with the percentages in their WT littermates. *AMPKα* cDKO mice also showed increased phospho-NF-κB p65 expression relative to their WT littermates at the age of 9 months (Fig. 6e,f; 1-way ANOVA; Sham, p = 0.047; DMM, p = 0.008; Unpaired *t*-test; Ageing, p < 0.001). We then analysed a chondrocyte apoptosis marker (TUNEL-positive cells) that was increased in *AMPKα* cDKO mice compared with their WT littermates 2 weeks post-DMM and at 9 months old (Fig. 7a,b; 1-way ANOVA; DMM, p = 0.002; Unpaired *t*-test; Ageing, p < 0.001). Regarding Adamts4, Adamts5, and Timp3 levels, no significant differences were observed between *AMPKα* cDKO and control mice (Supplementary Fig. S4a–f).

## Discussion

Multiple lines of evidence have suggested that AMPK could be a promising therapeutic target for OA given that AMPK activation in chondrocytes led to anti-catabolic and anti-apoptotic effects *in vitro*^8^,^9^, and AMPK imparts protection in an OA animal model^11^,^12^,^13^. Moreover, decreased AMPK activity was also detected in OA cartilage^8^,^9^. Therapies targeting AMPK are a good strategy for OA therapy and are attracting increasing attention^14^,^15^,^16^. However, there is still a lack of genetic evidence to understand the accurate role of AMPK in the homeostasis of adult articular cartilage. We investigated the direct effect of AMPK on adult articular cartilage maintenance using mice in which *AMPK* was deleted specifically in chondrocytes during the adult stage. Loss of *AMPK* aggravated the OA phenotype mainly via upregulating chondrocyte MMP-3 and MMP-13 levels and apoptosis.

Dual deficiency of *AMPKα1* and *AMPKα2* leads to embryonic lethality^21^. In this study, we found that the cartilage of *AMPKα* cDKO mice was indistinguishable at 10 weeks of age. The levels of knee joint damage of *AMPKα1* cKO, *AMPKα2* cKO or *AMPKα* cDKO mice were significantly increased compared with those in the respective WT littermates after DMM surgery. The greatest level of knee joint damage appeared in *AMPKα* cDKO mice. Both AMPKα1 and AMPKα2 catalytic subunits are essential for full AMPK activity in mouse chondrocytes, which is consistent with a previous report^8^. In contrast, a recent study showed that no significant difference was observed in cartilage degradation between *AMPKα1* cKO mice and control mice after 8 weeks of OA induction^17^. We note that the anterior cruciate ligament transection (ACLT) model was adopted in their study; however, this model has been suggested to be unsuitable for OA studies in mice given the development of severe OA and the high surgical proficiency required^18^.

AMPKα activity in articular chondrocytes is significantly decreased in ageing mice^9^. Thus, ageing-dependent reduction of AMPK activity might correlate with ageing-associated articular cartilage damage. We showed that severe cartilage damage of the knee joint was present in 12-month-old *AMPKα* cDKO males. Our data strongly suggest that AMPK activity is required for preventing articular cartilage degeneration during mouse ageing. Surprisingly, similar to WT mice, only mild damage in articular cartilage was observed in 12-month-old *AMPKα* cDKO females. In fact, in OA-susceptible mouse strains, males were more affected than females^22^, a finding that also holds for surgery-induced OA^18^. In contrast, epidemiological studies have demonstrated that the prevalence and severity of OA are significantly increased in women, especially after menopause, compared with those in men^23^,^24^. Currently, little is known regarding the sex-specific expression of AMPK. The difference between male and female mice may be further enhanced by the changes of sex hormones, and the mechanisms responsible for the sexual dimorphism of the chondroprotective role of AMPK remain to be determined.

An imbalance between chondrocyte anabolism and catabolism is the underlying mechanism of OA pathogenesis. The chondroprotective effect mediated by AMPK activation is associated with suppression of MMP gene expression^8^,^9^,^11^,^25^, and the modulation of NF-κB signalling has been suggested to be responsible for inhibiting IL-1β-induced expression of the MMP-13 gene in chondrocytes^19^,^20^. MMP-13 plays a key role in collagen matrix degradation^26^,^27^. Here, we showed that AMPK deficiency led to elevated MMP-13 expression in chondrocytes after IL-1β stimulation, which was associated with accelerated cartilage degeneration. Moreover, ablation of AMPK led to an increased phosphorylation level of the NF-κB p65 subunit in primary chondrocyte cultures. The findings obtained from these *in vivo* assessments support the *in vitro* results obtained in the present study. MMP-13 and phospho-NF-κB p65 expression levels were increased in the articular cartilage of *AMPKα* cDKO mice compared with those of WT mice 2 weeks post-DMM and at the age of 9 months.

Increased chondrocyte apoptosis also occurs in articular cartilage in response to OA factors, such as mechanical stress and ageing^28^,^29^. AMPK activators could suppress injury-induced bovine knee chondrocyte and sodium nitroprusside (SNP)-stimulated rat chondrocyte apoptosis *in vitro*^9^,^11^. Moreover, AMPK activators exhibited an anti-apoptotic effect on articular cartilage in a surgically induced rat OA model^11^. In this study, we first reported that advanced OA progression and increased apoptotic chondrocytes were present in *AMPKα* cDKO mice 2 weeks post-DMM and at 9 months. Together, these observations suggest that the excessive apoptotic process of chondrocytes might, in part, account for the accelerated OA progression in *AMPKα* cDKO mice.

In addition to articular destruction, we also observed increased synovial inflammation in 12-month-old male *AMPKα* cDKO mice. The increased synovitis score was likely because AMPK is a key anti-inflammatory agent. As an agonist of AMPK, simvastatin plays a beneficial role in inflammatory arthritis via up-regulation of SIRT1/FOXO3a signalling^30^. However, we could not observe any differences in the synovitis scores between AMPK mutant mice and their WT littermates at 2, 4 and 8 weeks post-DMM because this DMM model did not show appreciable synovitis^18^. The implication of AMPK in synovial inflammation requires further studies in which OA models with more synovitis are used.

In summary, we demonstrated that cartilage-specific deletion of *AMPKα* during the adult stage resulted in accelerated OA progression in a mouse model. Given that pharmacological activators, nutraceuticals, caloric restriction and exercise activate AMPK, future research on promoting AMPK activity in cartilage will lead to novel and effective therapeutic strategies for OA^5^,^31^,^32^,^33^,^34^.

## Methods

### Ethics statement

All animal procedures and protocols were approved by the Institutional Animal Care and Use Committee (IACUC) of the Model Animal Research Center of Nanjing University (Animal Care and Use Protocol Permit Number: JQ04). All surgeries were performed under anaesthesia, and all efforts were made to minimize suffering. All of the subsequent methods and analyses were performed in accordance with the relevant guidelines and regulations.

### Animals

C57BL/6-*AMPKα1*^*flox/flox*^ mice (Stock No: 014141, Jackson Laboratory) and C57BL/6-*AMPKα2*^*flox/flox*^ mice (Stock No: 014142, Jackson Laboratory) were kindly provided by Professor Shuai Chen (Nanjing University, Nanjing, China), who obtained these mice from Jackson Laboratories (Bar Harbor, ME, USA). Professor Minghao Zheng (University of Western Australia, Perth, Australia) kindly provided C57BL/6-*Col2a1-CreER*^*T2*^ mice^35^, which were generated in the laboratory of Professor Di Chen (Rush University Medical Center, Chicago, Illinois). *Col2a1-CreER*^*T2*^; *AMPKα1*^*flox/flox*^ and *Col2a1-CreER*^*T2*^; *AMPKα2*^*flox/flox*^ mice were generated by crossing *AMPKα1*^*flox/flox*^ and *AMPKα2*^*flox/flox*^ mice with *Col2a1-CreER*^*T2*^ mice, respectively. *Col2a1-CreER*^*T2*^; *AMPKα1*^*flox/flox*^*α2*^*flox/flox*^ mice were generated by crossing *Col2a1-CreER*^*T2*^; *AMPKα1*^*flox/flox*^ mice with *AMPKα2*^*flox/flox*^ mice. *AMPKα1*^*flox/flox*^, *AMPKα2*^*flox/flox*^ and *Cre* transgene were genotyped by PCR. *AMPKα1* primer sequences: upper primer, 5′-CCCACCATCACTCCATCTCT-3′ and lower primer, 5′-AGCCTGCTTGGCACACTTAT-3′. *AMPKα2* primer sequences: upper primer, 5′-GCAGGCGAATTTCTGAGTTC-3′ and lower primer, 5′-TCCCCTTGAACAAGCATACC-3′. *Cre* transgene primer sequences: upper primer, 5′-GCCTGCATTACCGGTCGATGC-3′ and lower primer, 5′-CAGGGTGTTATAAGCAATCCC-3′. The cartilage-specific *AMPKα1* cKO, *AMPKα2* cKO and *AMPKα* cDKO mice were injected intraperitoneally (IP) at age 8 weeks with TM (0.1 mg/g body weight, T5648, Sigma) daily for 5 days. The efficiency of *Cre* transgene recombination in the cartilage of these adult mice was evaluated as previously reported^36^,^37^. The Cre-negative WT littermates were also IP injected with the same dosage of TM and were used as controls. All mice in the OA studies were caged in groups (n = 3–6 mice per cage), maintained under 12-hour light/dark cycle and allowed free access to water and a standard rodent chow.

### RNA isolation

Articular cartilage was obtained from 10-week-old *AMPKα1* cKO, *AMPKα2* cKO, *AMPKα* cDKO mice and their WT littermates using a scalpel blade under a surgical microscope as described previously (n = 6/group)^38^. The dissected cartilage was immediately snap-frozen in liquid nitrogen and stored at −80 °C until RNA extraction. The cartilage was finely ground using a liquid nitrogen-chilled mortar and pestle followed by TRIzol extraction.

### Surgery-induced and ageing-associated OA model

The total number of mice used in this study was 366. The DMM-induced OA mouse model was generated in the right knees of 10-week-old *AMPKα1* cKO, *AMPKα2* cKO, *AMPKα* cDKO males and their corresponding WT littermates as previously described (n = 10–12/group)^18^. Briefly, after anaesthesia with 100 mg/kg ketamine combined with 5 mg/kg xylazine, the medial meniscotibial ligament that anchored the medial meniscus to the tibial plateau of the right knee was transected. The contralateral left knee joint was sham operated with medial capsulotomy only. Only male mice were used because of sex-related differences in the murine DMM surgical model^39^. Two, four or eight weeks later, the mice were sacrificed, and knee joints were harvested for histologic analyses.

For the ageing-associated model of OA, *AMPKα1* cKO, *AMPKα2* cKO, *AMPKα* cDKO mice and their WT littermates were sacrificed at the ages of 9 and 12 months (n = 5–11/group), and their right knees were used for histological analysis of spontaneous OA development.

### Histology

Whole freshly dissected mouse knee joints were fixed in 4% paraformaldehyde in 0.1 M phosphate-buffered saline (PBS) overnight at 4 °C, decalcified with 0.5 M EDTA (pH 7.4) for 1 week and embedded in paraffin. For quantification of instability-induced and ageing-associated spontaneous cartilage lesions, serial frontal sections (5 μm thick) were cut, with 3 to 4 sections per slide. Each paraffin block yielded 50 to 60 slides. Every sixth slide was stained with Safranin O/Fast Green (~10 slides per joint) or haematoxylin and eosin (H&E) staining (~10 slides per joint). The histological OA grade was evaluated using the Osteoarthritis Research Society International (OARSI) recommended 0 to 6 scoring system^40^. Articular surfaces within each section (the medial femoral condyle and the medial tibial plateau) were graded separately. In addition, synovial inflammation was determined by H&E staining, and synovitis was scored (grade 0–3) as previously described^41^. Osteophyte development was identified by Safranin O/Fast Green, and osteophyte maturity was determined (grade 0–3) as previously described^42^. All slides were evaluated by two independent investigators (SZ and WLL) in a blinded manner. The highest histopathology score among all slides was selected for the severity of cartilage destruction, synovial inflammation and osteophyte maturity. The histopathology scores of the two observers were averaged for each mouse prior to the statistical analyses.

### Chondrocyte primary cell culture

Mouse knee articular cartilage (femoral chondyle and tibial plateau) isolated from 5-day-old *Col2a1-CreER*^*T2*^; *AMPKα1*^*flox/flox*^*α2*^*flox/flox*^ mice and their *Cre*-negative littermates was enzymatically digested with 1% collagenase II (Gibco) to obtain primary chondrocytes as previously described^43^. Chondrocytes were seeded on a 10-cm dish at a density of 5 × 10^5^ cells per dish. On culture day 3, cells were treated with 4OH-TM (1 μM, H7904, Sigma) or vehicle for 48 hours. After reaching 80% confluence, cells were detached and plated in 6-well plates (5 × 10^5^/well). Chondrocytes were seeded 24 h prior to starving in 1:1 Dulbecco’s modified Eagle’s medium and Ham’s F-12 (DMEM/F12) containing 0.1% foetal bovine serum (FBS) for 12 h. Cells were then treated with IL-1β (10 ng/ml; R & D Systems) or vehicle for 24 h followed by RNA or protein extraction. Only the first passage cells were used for assays.

### Real-time reverse transcriptase-polymerase chain reaction (q-PCR) and Western blotting

Q-PCR and Western blotting were performed as described previously^44^. Primers names and sequences for q-PCR are listed in Supplementary Table 1. Total RNA from articular cartilage or murine chondrocytes was isolated using TRIzol reagent (Invitrogen) followed by reverse transcription using the PrimeScript RT Reagent Kit according to the manufacturer’s protocol (TaKaRa). Q-PCR was performed in an ABI Step One Plus instrument (Applied Biosystems) using SYBR Green PCR Master Mix (Thermo). Gene expression levels were defined using the 2^−ΔΔCt^ method. All reactions were performed in triplicate, and data were normalized to the mouse β-actin (Actb) gene.

Proteins were lysed with RIPA lysis buffer (50 mM Tris–HCl, pH 7.4, 150 mM NaCl, 1 mM EDTA, 0.5% sodium deoxycholate, 1% Nonidet P40 and 0.1% SDS) containing protease and phosphatase inhibitors, incubated on ice for 20 min and cleared by centrifugation (12,000 rpm for 20 min at 4 °C). The protein content of the lysates was determined with a bicinchoninic acid (BCA) protein assay kit (Pierce) using bovine serum albumin (BSA) as the standard. Normalized volumes of samples (20 μg protein) were separated in 12 to 15% Tris-glycine gel and transferred to nitrocellulose membranes, which were blocked with 5% (w/v) milk for 1 h and probed with diluted antibodies overnight. Proteins were detected using infrared dye-coupled secondary antibodies (goat anti-rabbit IRdye800, goat anti-rabbit IRdye680, goat anti-mouse IRdye800 and goat anti-mouse IRdye680). Membranes were scanned, and western blotting results were quantified using an Odyssey Infrared Imaging System (Li-Cor). The following primary antibodies were used: anti-AMPKα (1:1000, Cell Signalling Technology), anti-phospho-NF-κB p65 (1:500, Affinity), anti-NF-κB P65 (1:500, Abcam), anti-GAPDH (1:10,000; Sigma-Aldrich).

### IF, IHC and TUNEL

Sections were deparaffinized by xylene and rehydrated. After antigen retrieval by incubating in a sodium citrate buffer (0.01 M, pH 6.0) for 10 min, slides were washed and deprived of endogenous peroxidase activity with 3% hydrogen peroxide for 15 min. Sections were then blocked with 1% bovine serum albumin (Sigma) in PBS for 1 h at room temperature and incubated with primary antibodies at 4 °C overnight. Subsequently, slides were washed and incubated with Alexa Fluor 488 goat anti-rabbit secondary antibodies (1:500, Invitrogen) or horseradish peroxidase-conjugated goat anti-rabbit secondary antibodies (1:500, Invitrogen) and then incubated with a Vectstain ABC kit (Vector Laboratories Inc.). Primary antibodies against the following proteins were used: AMPKα1 (1:100, Proteintech), AMPKα2 (1:100, Proteintech), type II collagen (Col2a1, 1:100, Boster), Sox9 (1:100, Millipore), matrix metalloproteinase-3 (MMP-3, 1:100, Proteintech), matrix metalloproteinase-13 (MMP-13, 1:100, Proteintech), phospho-NF-κB p65 (Ser536) (1:50, Affinity), Adamts4 (1:500, Affinity), Adamts5 (1:500, Abcam) and Timp3 (1:100, Proteintech). TUNEL staining was performed using the *In Situ* Cell Death Detection Kit according to the manufacturer’s recommendations (Roche). As negative controls, non-immune rabbit IgG of the same dilution was used instead of the primary antibodies. Nuclei were counterstained with DAPI (Invitrogen) in IF and were scanned using a Leica TCS SP5 laser confocal microscope. Sections were stained with a 3, 3′-diaminobenzidine (DAB, Vector Laboratories Inc.) followed by counterstaining with haematoxylin in IHC and photographed under a Leica light microscope. Anatomically equivalent slides from *AMPKα* cDKO mice and WT littermates were used for quantification of the number of positive chondrocyte cells for each antigen or TUNEL. Sections from 3 slides per animal were imaged. The numbers of total chondrocytes and immune-positive cells across the entire tibial plateau were determined using Image J software. The final results from individual animals were averaged and presented as the percentage of each antigen or TUNEL-positive cells.

### Statistical analysis

Data were assessed for approximation to the Gaussian distribution using the D’Agostino and Pearson omnibus test of normality. Distributions were considered to be Gaussian if the P-value for the null hypothesis was greater than 0.05. If data did not fit a Gaussian distribution, we then transformed the values by taking the square root. The transformed values approximated a Gaussian distribution. When multiple comparisons were performed, a Bonferroni *post-hoc* test was used to adjust for multiplicity. To derive the number of mice required, we performed power calculations with reference to our published study^45^. Unpaired two-tailed Student t test, 1-way analysis of variance (ANOVA) and 2-way ANOVA were used in this study. All data are expressed as means ± SD. P-values less than 0.05 were considered statistically significant. GraphPad Prism version 6 was used for statistical analysis.

## Additional Information

**How to cite this article:** Zhou, S. *et al*. AMPK deficiency in chondrocytes accelerated the progression of instability-induced and ageing-associated osteoarthritis in adult mice. *Sci. Rep.* **7**, 43245; doi: 10.1038/srep43245 (2017).

**Publisher's note:** Springer Nature remains neutral with regard to jurisdictional claims in published maps and institutional affiliations.

## Supplementary Material

Supplementary Information
