# Supplementary material for: AMPK deficiency in chondrocytes accelerated the progression of instability-induced and ageing-associated osteoarthritis in adult mice
Source: Sci Rep. 2017 Feb 22;7:43245. doi: 10.1038/srep43245 (PMC5320548; doi:10.1038/srep43245)
Supplement: Supplementary Information [file srep43245-s1.pdf]

Supplementary Information

AMPK deficiency in chondrocytes accelerated the progression of instability-induced and ageing-associated osteoarthritis in adult mice

Sheng Zhou[1], Wanli Lu[1], Liang Chen[2], Qiting Ge[2], Dongyang Chen[1], Zhihong Xu[1], Dongquan Shi[1], Jin Dai[1], Jianxin Li[3], Huangxian Ju[3], Yi Cao[4], Jinzhong Qin[2], Shuai Chen[2], Huajian Teng[2]* and Qing Jiang[1,2,3]*

Supplementary Figures

Figure S1. (a) Genotyping showing the presence of *Cre* transgene in *AMPKα1* conditional knockout (*AMPKα1* cKO), *AMPKα2* cKO, *AMPKα1α2* conditional double knockout (*AMPKα* cDKO) mice and its absence in their *Cre*-negative WT littermates; *AMPKα1* KO band was detected at 450bp, *AMPKα1* WT band at 334bp, *AMPKα2* KO band at 450bp, *AMPKα2* WT band at 341bp, and *Cre* band at 500bp.

Figure S2. Accelerated OA in *AMPKα1* conditional knockout (*AMPKα1* cKO) and *AMPKα2* cKO mice following destabilization of the medial meniscus (DMM). (a and c) Representative photographs of articular cartilage destruction in *AMPKα1* cKO, *AMPKα2* cKO mice and their WT littermates 2, 4 and 8 weeks post-DMM (n = 12/group). Sections were stained with Safranin O/Fast Green. Loss of uncalcified cartilage, a reduced number of chondrocytes, and alteration of the tidemark integrity (arrowheads) were observed in *AMPKα1* cKO and *AMPKα2* cKO mice. Scale bars =

100 μm. (b and d) The OARSI scores for the medial femoral condyle and the medial tibial condyle at 2, 4 and 8 weeks post-DMM in *AMPKα1* cKO, *AMPKα2* cKO mice and their WT littermates (n = 10/group). The OARSI scores for the medial femoral condyle of *AMPKα1* cKO and their WT littermates post-DMM were transformed by taking the square root of the values. After transformation, all groups of data approximate a Gaussian distribution. * $p < 0.05$. ** $p < 0.01$. NS = not significant.

Figure S3. The OARSI scores for the medial femoral condyle and the medial tibial condyle at 2, 4 and 8 weeks post-sham operation in *AMPKα1α2* conditional double knockout (*AMPKα* cDKO) (a), *AMPKα1* conditional knockout *(AMPKα1* cKO) (b), *AMPKα2* cKO mice (c) and their WT littermates. The OARSI scores for the contralateral medial femoral and tibial condyle of *AMPKα2* cKO and their WT littermates post-DMM were transformed by taking the square root of the values. After transformation all groups of data approximate a Gaussian distribution.* $p < 0.05$. ** $p < 0.01$. NS = not significant.

Figure S4. IHC analyses of surgically induced and ageing-associated OA. (a, c and e) Representative IHC images of Adamts4, Adamts5 and Timp3 in the medial tibial plateau in *AMPKα1α2* conditional double knockout (*AMPKα* cDKO) mice and their WT littermates 2 weeks post-sham operation and DMM or in mice at 9 months of age. Scale bars = 20 μm. Cellularity of section was confirmed with hematoxylin staining. (b, d, f) Quantifications of the percentage of Adamts4, Adamts5 and Timp3 are presented as percentages relative to cells stained for hematoxylin. (n = 6 / group) NS = not significant.

Table S1. List of primer names and sequences for q-PCR

Primer name	Sequences
Prkaa1 upper	CTTGACGTGGTGGGAAAAAT
Prkaa1 lower	CCACCATATGCCTGTGACAA
Prkaa2 upper	ACAGGCCATAAAGTGGCAGT
Prkaa2 lower	GCCCATGTTTGCAGATGTAG
Col2a1 upper	CAGGATGCCCGAAAATTAGGG
Col2a1 lower	ACCACGATCACCTCTGGGT
Aggrecan upper	CCTGCTACTTCATCGACCCC
Aggrecan lower	AGATGCTGTTGACTCGAACCT
Sox9 upper	AGTACCCGCATCTGCACAAC
Sox9 lower	ACGAAGGGTCTCTTCTCGCT
MMP-3 upper	ACATGGAGACTTTGTCCCTTTTG
MMP-3 lower	TTGGCTGAGTGGTAGAGTCCC
MMP-13 upper	TGTTTGCAGAGCACTACTTGAA
MMP-13 lower	CAGTCACCTCTAAGCCAAAGAAA
Adamts4 upper	ATGGCCTCAATCCATCCCAG
Adamts4 lower	AAGCAGGGTTGGAATCTTTGC
Adamts5 upper	GGAGCGAGGCCATTTACAAC
Adamts5 lower	CGTAGACAAGGTAGCCCACTTT
Timp3 upper	TTATCCCATTGGGGCATTTA
Timp3 lower	TTGCTGCCTTTGACTGATTG

Actb upper	GGCTGTATTCCCCTCCATCG
Actb lower	CCAGTTGGTAACAATGCCATGT

Figure S1

a

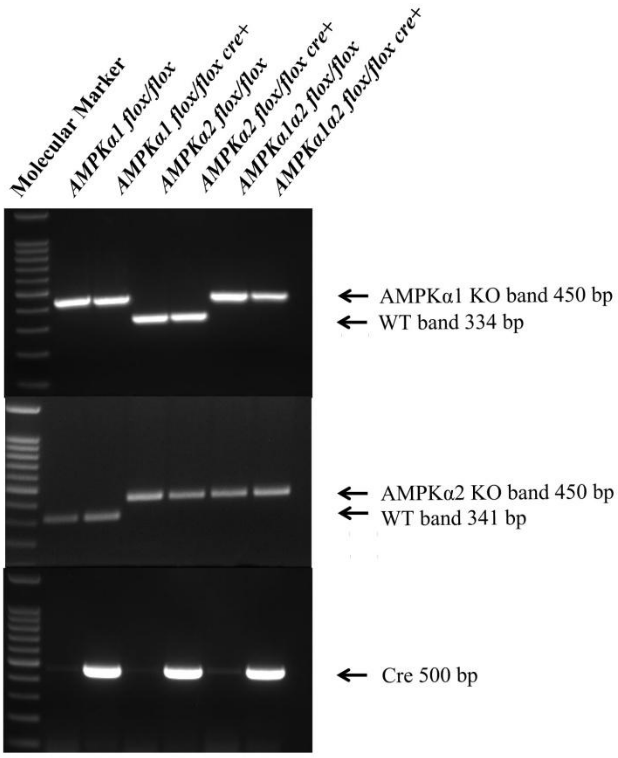
Molecular Marker
AMPKα1 flox/flox
AMPKα1 flox/flox cre+
AMPKα2 flox/flox
AMPKα2 flox/flox cre+
AMPKα1α2 flox/flox
AMPKα1α2 flox/flox cre+
AMPKα1 KO band 450 bp
WT band 334 bp
AMPKα2 KO band 450 bp
WT band 341 bp
Cre 500 bp

Figure S2

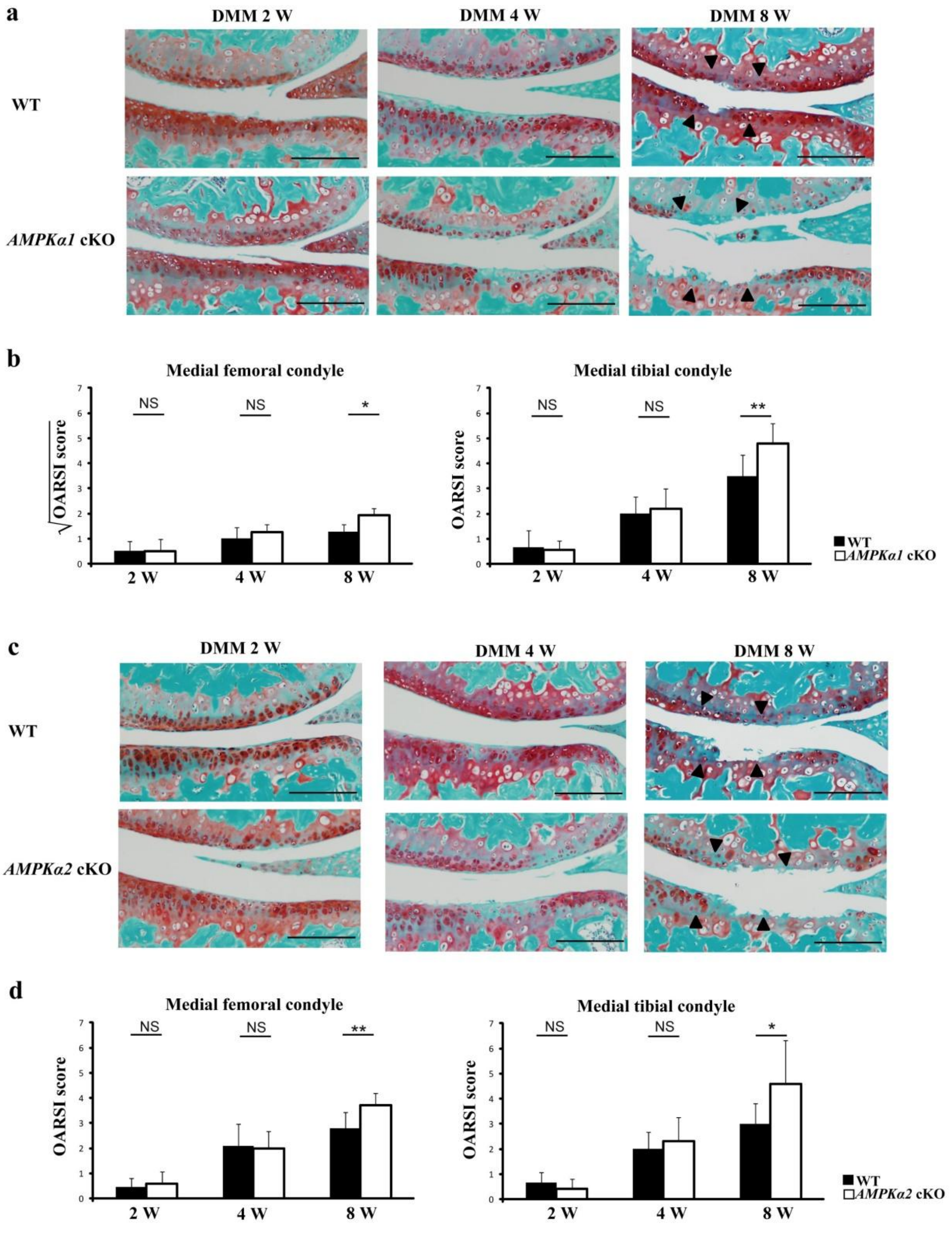
a
DMM 2 W
DMM 4 W
DMM 8 W
WT
AMPKα1 cKO
b
Medial femoral condyle
Medial tibial condyle
√OARSI score
OARSI score
NS
NS
*
NS
NS
**
2 W
4 W
8 W
WT
AMPKα1 cKO
c
DMM 2 W
DMM 4 W
DMM 8 W
WT
AMPKα2 cKO
d
Medial femoral condyle
Medial tibial condyle
OARSI score
NS
NS
**
NS
NS
*
2 W
4 W
8 W
WT
AMPKα2 cKO

Figure S3

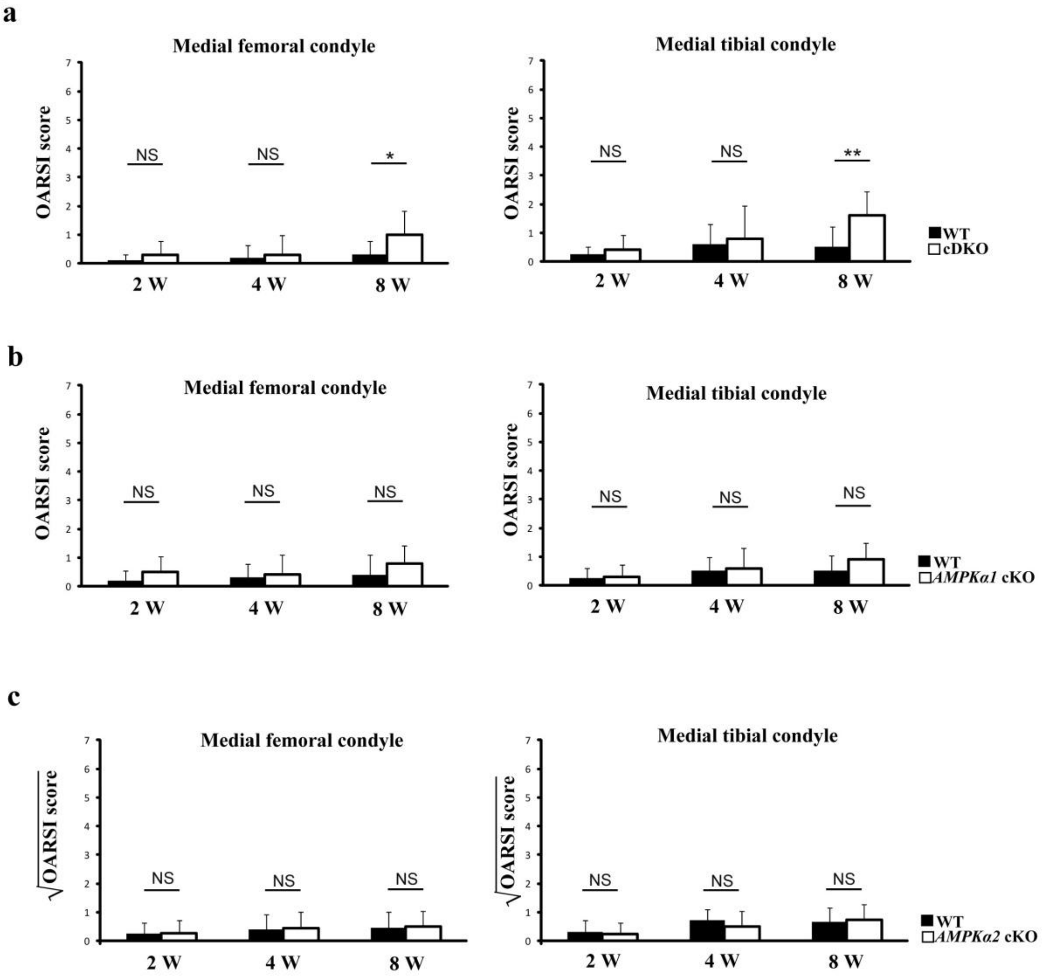
a
Medial femoral condyle
Medial tibial condyle
OARSI score
NS
NS
*
NS
NS
**
2 W
4 W
8 W
WT
cDKO
b
Medial femoral condyle
Medial tibial condyle
OARSI score
NS
2 W
4 W
8 W
WT
AMPKα1 cKO
c
Medial femoral condyle
Medial tibial condyle
√OARSI score
NS
2 W
4 W
8 W
WT
AMPKα2 cKO

Figure S4

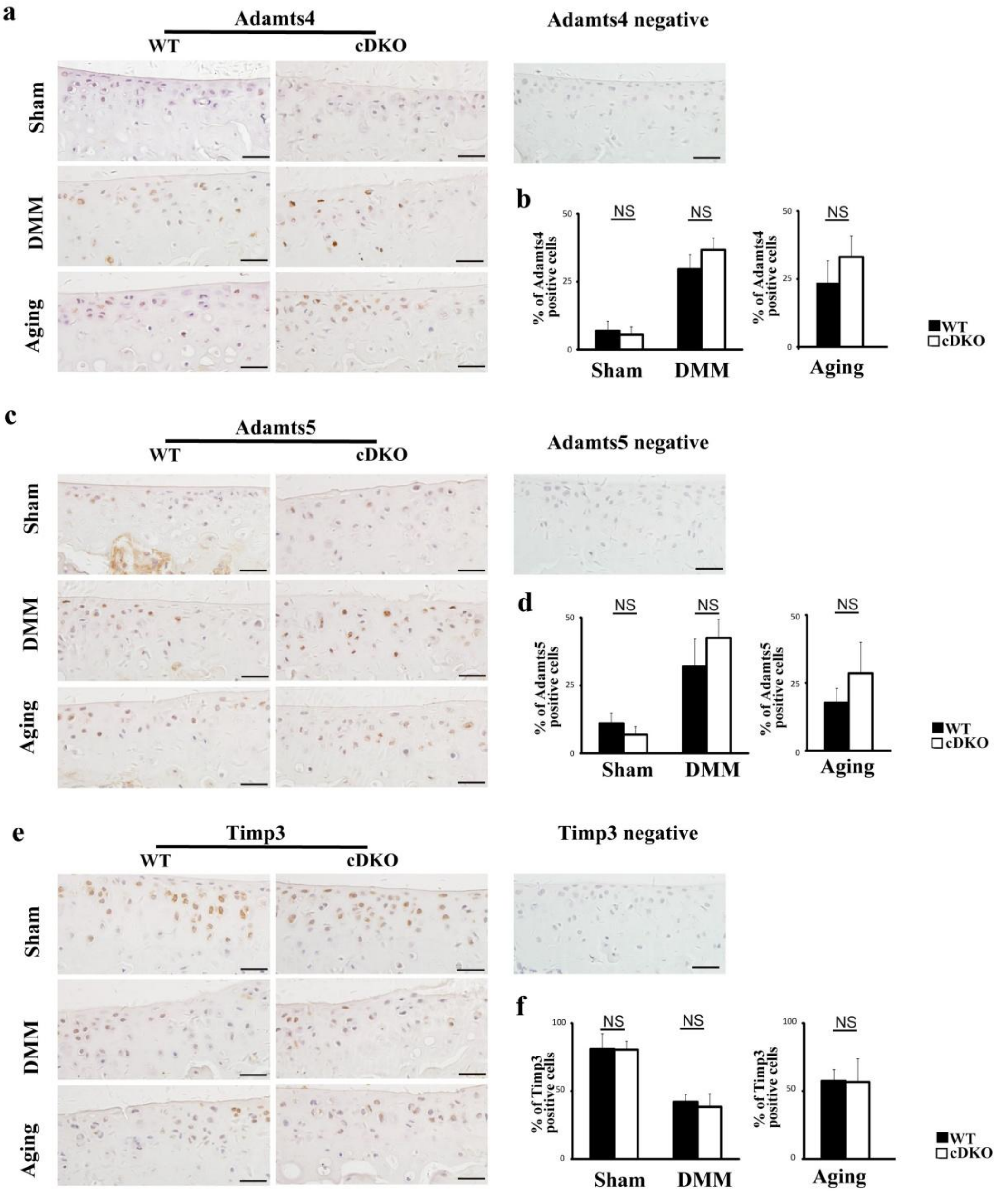

a
Adamts4
WT
cDKO
Adamts4 negative
Sham
DMM
Aging
b
NS
NS
NS
% of Adamts4 positive cells
50
25
0
Sham
DMM
Aging
WT
cDKO
c
Adamts5
WT
cDKO
Adamts5 negative
Sham
DMM
Aging
d
NS
NS
NS
% of Adamts5 positive cells
50
25
0
Sham
DMM
Aging
WT
cDKO
e
Timp3
WT
cDKO
Timp3 negative
Sham
DMM
Aging
f
NS
NS
NS
% of Timp3 positive cells
100
50
0
Sham
DMM
Aging
WT
cDKO
